# Intraoperative hemodynamics and risk of cardiac surgery‐associated acute kidney injury: An observation study and a feasibility clinical trial

**DOI:** 10.1111/1440-1681.13812

**Published:** 2023-08-07

**Authors:** Khin M. Noe, Andrea Don, Andrew D. Cochrane, Michael Z. L. Zhu, Jennifer P. Ngo, Julian A. Smith, Amanda G. Thrift, Johnny Vogiatjis, Andrew Martin, Rinaldo Bellomo, James McMillan, Roger G. Evans

**Affiliations:** ^1^ Cardiovascular Disease Program, Department of Physiology Biomedicine Discovery Institute, Monash University Melbourne Victoria Australia; ^2^ Department of Surgery School of Clinical Sciences at Monash Health, Monash University Melbourne Victoria Australia; ^3^ Department of Cardiothoracic Surgery Monash Health, Monash University Melbourne Victoria Australia; ^4^ Department of Cardiac Physiology National Cerebral and Cardiovascular Center Research Institute Osaka Japan; ^5^ Department of Medicine School of Clinical Sciences at Monash Health, Monash University Melbourne Victoria Australia; ^6^ Department of Critical Care University of Melbourne Melbourne Victoria Australia; ^7^ Department of Intensive Care, Austin Health Heidelberg Victoria Australia; ^8^ Pre‐clinical Critical Care Unit Florey Institute of Neuroscience and Mental Health, University of Melbourne Melbourne Victoria Australia; ^9^ Australian and New Zealand Intensive Care Research Centre Monash University Melbourne Victoria Australia; ^10^ Perfusion Services Pty Ltd Melbourne Victoria Australia

**Keywords:** arterial pressure, cardiopulmonary bypass, clinical perfusion, pump flow, systemic oxygen delivery

## Abstract

Targeting greater pump flow and mean arterial pressure (MAP) during cardiopulmonary bypass (CPB) could potentially alleviate renal hypoxia and reduce the risk of postoperative acute kidney injury (AKI). Therefore, in an observational study of 93 patients undergoing on‐pump cardiac surgery, we tested whether intraoperative hemodynamic management differed between patients who did and did not develop AKI. Then, in 20 patients, we assessed the feasibility of a larger‐scale trial in which patients would be randomized to greater than normal target pump flow and MAP, or usual care, during CPB. In the observational cohort, MAP during hypothermic CPB averaged 68.8 ± 8.0 mmHg (mean ± SD) in the 36 patients who developed AKI and 68.9 ± 6.3 mmHg in the 57 patients who did not (*p* = 0.98). Pump flow averaged 2.4 ± 0.2 L/min/m^2^ in both groups. In the feasibility clinical trial, compared with usual care, those randomized to increased target pump flow and MAP had greater mean pump flow (2.70 ± 0.23 vs. 2.42 ± 0.09 L/min/m^2^ during the period before rewarming) and systemic oxygen delivery (363 ± 60 vs. 281 ± 45 mL/min/m^2^). Target MAP ≥80 mmHg was achieved in 66.6% of patients in the intervention group but in only 27.3% of patients in the usual care group. Nevertheless, MAP during CPB did not differ significantly between the two groups. We conclude that little insight was gained from our observational study regarding the impact of variations in pump flow and MAP on the risk of AKI. However, a clinical trial to assess the effects of greater target pump flow and MAP on the risk of AKI appears feasible.

## 1 INTRODUCTION

Acute kidney injury (AKI) is a common and serious postoperative complication of cardiac surgery requiring cardiopulmonary bypass (CPB). Globally, AKI occurs after approximately 22% of the estimated two million such cardiac procedures performed yearly, although reported incidence has varied widely in the published literature.^1^ Patients who develop AKI have a greatly increased risk of both short‐term and long‐term mortality^1^ and also chronic kidney disease.^2^, ^3^

Renal hypoxia may be a critical pathological feature of multiple forms of AKI,^4^ including cardiac surgery‐associated AKI (CSA‐AKI).^5^ The kidney,^6^, ^7^ and particularly the renal medulla,^8^, ^9^ appears to be susceptible to hypoxia during CPB. There is also the potential for renal injury during reperfusion and re‐oxygenation.^5^, ^7^ Furthermore, intraoperative renal hypoxia, as assessed indirectly by near‐infrared spectroscopy^10^, ^11^ or measurement of urinary oxygen tension,^12^, ^13^, ^14^, ^15^, ^16^ predicts postoperative AKI. Low intraoperative urinary oxygen tension is also associated with poorer 12‐month outcomes after cardiac surgery on CPB.^17^ Thus, efforts to alleviate renal hypoxia during CPB could mitigate the risk of CSA‐AKI. Increasing pump flow or target arterial pressure during CPB,^8^ or increasing both variables simultaneously,^18^, ^19^ can increase renal oxygen delivery and alleviate renal tissue hypoxia in experimental CPB. Increased pump flow and arterial pressure also appear to be associated with increased urinary PO_2_ in patients during CPB.^20^ Thus, the relatively simple intervention of increasing pump flow and arterial pressure could reduce the risk of CSA‐AKI. Increased pump flow or arterial pressure during CPB also has the potential for adverse effects on the kidney, such as increased hemolysis with hematuria and the nephrotoxic effects of increased concentration of free haemoglobin in blood.^21^, ^22^

The current investigation comprised two phases. In the first, we performed an exploratory analysis of a single‐centre observational study of 93 patients undergoing cardiac surgery, with the outcome of CSA‐AKI assessed prospectively. The aim of the current analysis was to determine variations in usual care with respect to hemodynamic management and whether any systematic differences in hemodynamic management could be detected between patients who did and did not develop postoperative AKI. Having determined from these observations that patient management was relatively ‘homogeneous’ (i.e., standard protocols were followed so that there was little between‐patient variation in hemodynamic management during CPB), we then conducted a feasibility clinical trial to determine whether a clinical trial in which patients are randomized to higher target flow and mean arterial pressure (MAP) during CPB, or usual care, is feasible.

## 2 METHODS

### 2.1 Observational study

#### 2.1.1 Recruitment

The study was conducted with approval from the Human Research Ethics Committee (HREC) at Monash Health (Approval number: 12375B). Patients were recruited into the study when they attended the outpatients’ clinic or when in hospital (before their surgical procedure). All patients provided written informed consent before participation.

**FIGURE 1:**
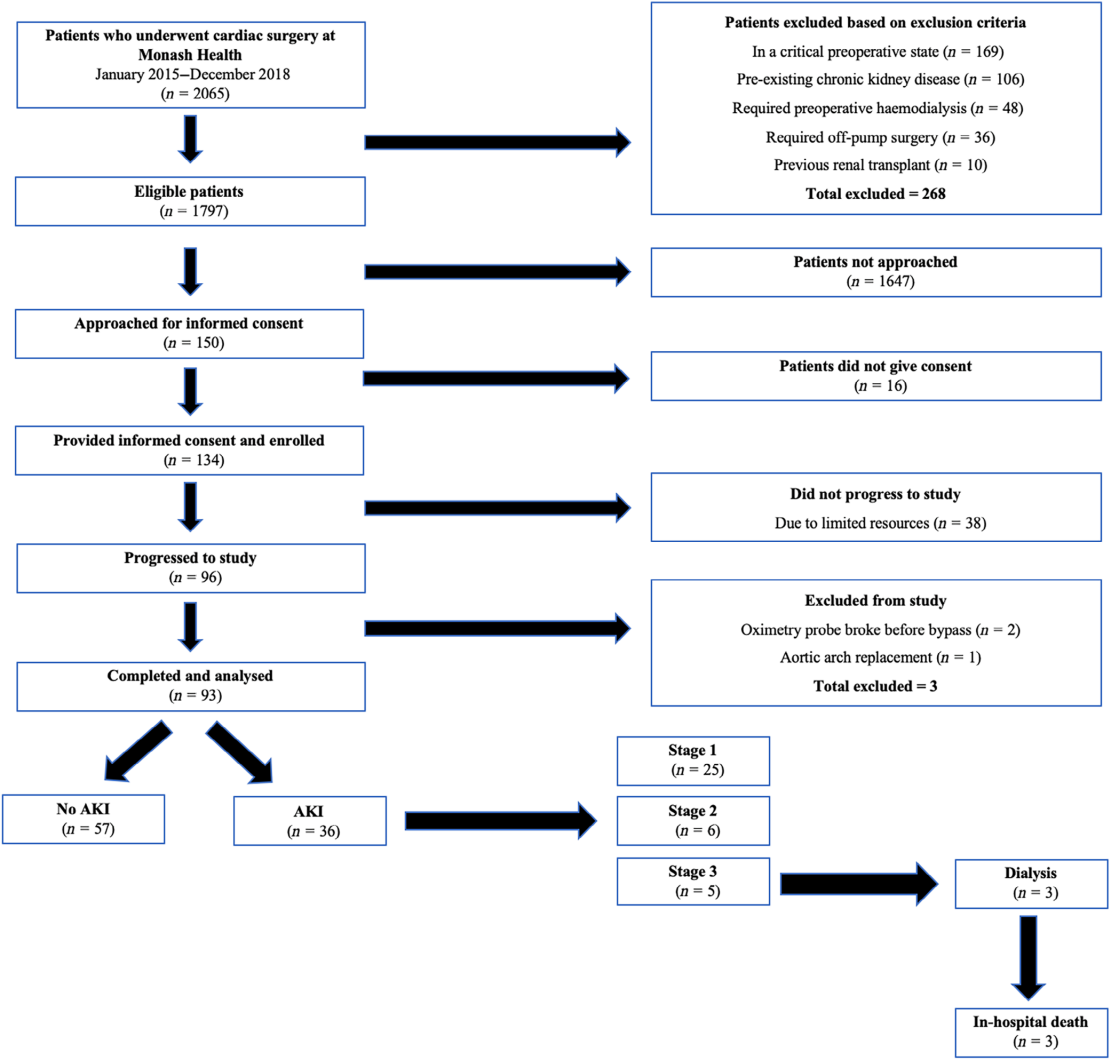
Recruitment of patients: observational study. Patients with a high risk of postoperative mortality, assessed using EuroScore II,^27^ were preferably selected over patients with a low risk of postoperative mortality. The study was originally designed and powered to investigate the utility of continuous measurement of urinary oxygen tension for predicting postoperative acute kidney injury. These analyses utilizing measurement of urinary oxygen tension have been reported previously.^13^, ^14^, ^16^, ^23^, ^24^, ^25^ Some patients who provided informed consent did not progress to the study because only one urinary oxygen monitor was available. Thus, only one patient could be studied at a time.

From January 2015 to December 2018, 93 patients undergoing cardiac surgery requiring CPB were recruited at Monash Health (Figure 1). The study was originally designed to investigate the potential of continuous measurement of urinary oxygen tension for assessing the risk of AKI during and after cardiac surgery; these analyses are now published.^13^, ^14^, ^16^, ^23^, ^24^, ^25^ A consequence of this original aim was that many patients could not be studied due to unavailability of the single oxygen monitor that was available to us. Consequently, the sample of patients was non‐consecutive. We recruited patients who were having coronary artery bypass graft (CABG) surgery, valve repair/replacement or combined CABG–valve repair procedures. Patients were excluded if they had preoperative chronic kidney disease (baseline SCr >200 μmol/L or estimated glomerular filtration rate [eGFR] <30 mL/min/1.73 m^2^), a confirmed preoperative diagnosis of AKI as defined by Kidney Disease Improving Global Outcomes (KDIGO) criteria,^26^ were undergoing acute or long‐term haemodialysis, had received a kidney transplant, were undergoing off‐pump surgery or were unable or unwilling to provide written informed consent. Patients with a higher EuroSCORE‐II (risk of early postoperative mortality)^27^ were recruited when possible for the observational study.

#### 2.1.2 Standard intraoperative procedures

Standard institutional protocols were followed. In brief, after pre‐medication with oral temazepam (10 mg) and oxycodone (10 mg), radial arterial and central venous catheters were inserted for standard monitoring and vascular access. A Swan‐Ganz catheter was inserted to permit monitoring of pulmonary arterial pressure and cardiac output. Intravenous midazolam (2–5 mg), fentanyl (5–10 μg/kg), pancuronium (0.1 mg/kg) and a titrated dose of propofol was used for induction. After intubation, anaesthesia was maintained with sevoflurane (1–2% v/v) in 60% oxygen and propofol (5–8 mg/kg h^−1^). All patients received tranexamic acid (50 mg/kg) and cefazolin (2 g). The trigger for red blood cell transfusion was typically ≤80 g/L. Target mean arterial pressure (60–80 mmHg) before, during and after CPB was achieved by administration of metaraminol and/or norepinephrine.

Via median sternotomy, cardiopulmonary bypass (CPB) was established by cannulation of the ascending aorta and two‐stage venous cannulation in the right atrium. To achieve an activated clotting time >450 s, heparin (300 IU/kg) was administered prior to cannulation. The circuit included a 40‐μm arterial line filter, a roller pump (Stöckert SIII; Stöckert Instrumente, Munich, Germany) and a hard‐shell membrane oxygenator (Synthesis; Sorin Biomedica, Modena, Italy), primed with 1600 mL of Hartmann's solution, 5000 IU of heparin and 1 g of cefazolin. During CPB, the target pump flow (non‐pulsatile) was 2.2 to 2.4 L min^−1^ m^−2^, the target haematocrit was 20 to 30%, the target venous oxygen saturation was >75%, and the target body temperature during the hypothermic phase of CPB was 34°C. Intermittent anterograde or retrograde cardioplegia consisted of four parts crystalloid (St. Thomas Solution No. 2; Hospira, Illinois, USA) and one‐part patient blood. Target nasopharyngeal temperature for separation from bypass was 36 to 36.5°C with a rewarming rate of approximately 0.5°C per minute. After weaning from CPB and de‐cannulation, heparin was reversed with protamine (1 mg for each 100 IU loading dose of heparin).

#### 2.1.3 Recording of intraoperative hemodynamic variables

At 5‐min intervals, a dedicated researcher manually recorded the current value of all data available from patient monitors within the operating theatre. Body temperature (°C), MAP (mmHg) and oxygen saturation of arterial haemoglobin (S_A_O_2_, %) were measured throughout the entire operation. Systolic and diastolic arterial pressure (mmHg), systolic, diastolic and mean pulmonary arterial pressure (mmHg), heart rate (beats/min), end‐tidal PCO_2_ (mmHg) and central venous pressure (mmHg) were all recorded when the patient was not on CPB. Variables measured only during CPB were: pump flow (L/min/m^2^), arterial blood haemoglobin concentration (g/L), mixed venous oxygen saturation (S_v_O_2_; %), systemic oxygen delivery (DO_2_, ml/min/m^2^), systemic oxygen consumption (VO_2_, mL/min/m^2^), systemic fractional oxygen extraction and the partial pressures of oxygen and carbon dioxide in arterial blood (P_A_O_2_ and P_A_CO_2_; mmHg).

For analytic purposes, the data were divided into five major epochs. These were the preoperative period (from induction of anaesthesia to the time of the first incision), pre‐CPB (the period from the first incision to just before the initiation of CPB), CPB‐Hypothermic (the period on CPB before rewarming), CPB‐Rewarming (the period from commencing rewarming until weaning from CPB began) and post‐CPB (the period from termination of CPB to the end of the surgical procedure).

#### 2.1.4 Definition of acute kidney injury

Acute kidney injury was defined as an increase in serum creatinine concentration of ≥26.5 μmol/L (0.3 mg/dL) within 48 h and/or ≥ 50% elevation from baseline within 5 days of the surgical procedure. This modified version of the KDIGO criteria^26^ did not include consideration of urine flow because the use of urine flow criteria can result in overdiagnosis of CSA‐AKI.^28^, ^29^ Furthermore, urine flow in the ICU was approximated rather than measured with precision, so the data we have for urine flow may be unreliable. We also made no adjustments to the serum creatinine measurements in patients who received intraoperative hemofiltration or dialysis because we could not access these patients’ data.

#### 2.1.5 Statistical analyses

##### Preoperative data

Categorical variables are presented as number of patients (%). Continuous variables and clinical risk scores are presented as the median (25th percentile–75th percentile) because they commonly failed the Shapiro–Wilk normality test. Differences between patients who did and did not develop AKI were assessed by χ^2^‐test (categorical variables; or Fisher's exact test for counts <5) or the Mann–Whitney *U*‐test (continuous variables).

##### Intraoperative data

Continuous variables are expressed as mean ± standard deviation. Missing values were imputed by conditional mean imputation^30^ in cases when data for only a single epoch was missing for a single patient within a diagnostic group. Otherwise, the entire patient was excluded for the analysis of that variable. Intraoperative data were first subjected to repeated measures analysis of variance with the application of the Greenhouse–Geisser correction to control the risk of type 1 error.^31^ The analysis included the between‐subjects factor ‘Group’ (whether patients did or did not develop AKI) and the within‐subjects factor ‘Epoch’ and their interaction. When the main effect ‘Group’ or the ‘Group*Epoch’ interaction term was found to be statistically significant, Student's unpaired *t*‐test was used to make specific comparisons between patients who did and did not develop AKI at each epoch. When either the main effect ‘Epoch’ or the ‘Group*Epoch’ interaction term was found to be statistically significant, Student's paired *t*‐test was used to make specific comparisons between each individual epoch and the baseline (preoperative) epoch within each diagnostic category.

Two‐tailed *p* ≤ 0.05 was considered statistically significant.

### 2.2 Feasibility trial

#### 2.2.1 Recruitment

From September 2019 to September 2021, 20 patients were recruited into a single‐centre trial to assess the feasibility of a large‐scale trial of increasing pump flow and MAP during CPB. During the period of recruitment, the trial was temporarily suspended on multiple occasions due to restrictions associated with the COVID‐19 pandemic.

The trial was registered on the Australian and New Zealand Clinical Trials Register (ACTRN12619000128190). Registration was for a sample size of 400, powered to detect a difference in the incidence of AKI between the intervention (11.5%) and usual care (22.4%) arms. Due to insufficient funds and delays caused by the COVID‐19 pandemic, the trial was terminated in September 2021 to allow analysis as a feasibility study. The protocol was approved by the Human Research Ethics Committee at Monash Health (HREC approval number 46510). All patients provided written informed consent.

Inclusion criteria were similar to those for the observational study. Patients were excluded if (i) their expected duration of CPB was less than 90 min, (ii) their expected lowest core body temperature during CPB was to be less than 32°C, (iii) they had severe preoperative chronic kidney disease (serum creatinine >265 μmol/L), (iv) they had pre‐existing AKI as defined by KDIGO criteria,^26^ (v) they had been receiving acute or long‐term haemodialysis, (vi) they had received a kidney transplant, (vii) they were unable or unwilling to provide written informed consent, or (viii) off‐pump surgery was planned.

#### 2.2.2 Patient management and randomization

All surgical procedures and patient management followed the standard protocols at Monash Health, which were the same as for the observational study.^13^, ^14^, ^16^ The intervention was a target pump flow of 2.9 L/min/m^2^ and target mean arterial pressure of ≥80 mmHg. Patients were randomized to the intervention or usual care group after induction of anaesthesia. Randomization was blocked by Mehta score^32^ (risk of requirement of postoperative dialysis) ≥ or <0.7% using Research Electronic Data Capture (RedCap) software.^33^ The chief barrier to achieving higher pump flow is the level of venous return, which determines the volume in the blood reservoir in the CPB circuit. The volume within this reservoir can be increased by adding donor blood (leading to hemoconcentration) or crystalloid (leading to haemodilution). To avoid such changes in blood oxygen carrying capacity, perfusionists were instructed to refrain from adding extra blood or crystalloid to the pump reservoir to achieve the target pump flow in the intervention group. Instead, perfusionists were asked to target the greatest flow, up to a maximum of 2.9 L/min/m^2^, that could be achieved by standard volume management. Metaraminol was administered as required by the perfusionist to achieve the target MAP.

#### 2.2.3 Data collection

All clinical and surgical data were collected following the same approach as for the observational study.

#### 2.2.4 Blinding procedure

The trial was double‐blinded in that the patients were unaware of their treatment allocation, and we attempted to blind most of the medical staff involved in the patient's care. That is, all staff in the operating room were blinded to the patients’ treatment allocation except the perfusionists and the dedicated researcher conducting the trial. It was impossible to conceal the treatment allocation from the perfusionists as they were responsible for operating the CPB machine and, therefore, administered the intervention. The awareness of the patients’ treatment allocation by the rest of the team was assessed at the end of the operation. After each case, the lead surgeon, the anaesthetist and the lead surgical nurse were asked to identify or guess the allocation of the patient.

#### 2.2.5 Feasibility outcomes

The main outcomes for the trial were (i) recruitment, (ii) pump flow and MAP during CPB, (iii) effectiveness of blinding and (iv) survival to discharge and 90 days after surgery.

#### 2.2.6 Statistical analysis

Data were analysed and are presented in the same manner as for the observational study. However, in this case, the between‐subjects factor in repeated measures analysis of variance was treatment allocation (usual care vs intervention). As for the observational study, when significant main effects or interaction terms were statistically significant, post‐hoc comparisons were made between the two treatment allocations at each epoch using Student's unpaired *t*‐test. The effectiveness of blinding was assessed using binomial probability. Two‐tailed *p* ≤ 0.05 was considered statistically significant.

## 3 RESULTS

### 3.1 Observational study

#### 3.1.1 Patient characteristics

Postoperative AKI was diagnosed in 38.7% (36/93) of patients (Figure 1). This relatively high incidence most likely reflects our intentional bias in the recruitment of high‐risk patients based on EuroScore II (see Methods). Patients who developed AKI had more severe preoperative heart failure (New York Heart Association classification ≥3) and were more likely to have had multi‐valve surgery than patients who did not (Table 1). Furthermore, preoperative serum creatinine (SCr) was 12% greater, and the eGFR was 15% less in patients who developed AKI than in those who did not. These observations likely reflect that the severity of preoperative heart failure, the complexity of surgery and preoperative renal dysfunction are established risk factors for CSA‐AKI.^34^ Patients who developed AKI had a higher Mehta score,^32^ but there were no significant differences in the Cleveland Clinic Score^35^ or EuroScore II^27^ between the two groups of patients.

**TABLE 1:** Baseline characteristics in the observational study.

Characteristic	AKI (*n* = 36)	No AKI (*n* = 57)	*p*
Categorical variables			
Gender, female	7 (19.4)	11 (19.3)	0.99
Type of surgery			
Isolated CABG	19 (52.8)	35 (61.4)	0.53
Single valve	5 (13.9)	12 (21.1)	0.38
Single valve + CABG	7 (19.4)	10 (17.5)	0.82
>1 valve	5 (13.9)	0 (0.0)	**0.004**
Status of surgery			
Elective	26 (72.2)	45 (78.9)	0.46
Urgent	9 (25.0)	12 (21.1)	0.69
Emergency	1 (2.8)	0 (0.0)	0.21
Risk factors and comorbidity			
Hypertension	32 (88.9)	44 (77.2)	0.16
Hypercholesterolemia	29 (80.6)	41 (71.9)	0.35
Angina	21 (58.3)	35 (61.4)	0.52
Diabetes mellitus	16 (44.4)	21 (36.8)	0.47
Smoking history	22 (61.1)	31 (54.4)	0.52
Myocardial infarct	10 (27.8)	22 (38.6)	0.29
NYHA ≥ III	14 (38.9)	10 (17.5)	**0.02**
Arrhythmia	10 (27.8)	11 (19.3)	0.34
Congestive cardiac failure	7 (19.4)	9 (15.8)	0.65
Chronic lung disease	6 (16.7)	3 (5.3)	0.07
Peripheral vascular disease	4 (11.1)	5 (8.8)	0.71
Cerebrovascular disease	4 (11.1)	5 (8.8)	0.71
Estimated LVEF <30%	4 (11.1)	2 (3.5)	0.59
Redo sternotomy	3 (8.3)	3 (5.3)	0.56
Continuous variables			
Age, years	70.8 (61.8–77.8)	71.8 (65.2–77.1)	0.35
Body mass index, kg/m^2^	29.8 (26.5–33.1)	28.1 (25.3–31.3)	0.19
Baseline SCr, μmol/L	86.3 (76.0–106.6)	77.0 (66.0–88.0)	**0.01**
Baseline eGFR, mL/min/1.73 m^2^	71.0 (57.8–85.9)	83.5 (72.2–92.5)	**0.02**
Clinical risk scores			
Cleveland clinic score, % risk^35^	1.8 (0.4–1.8)	1.8 (0.4–1.8)	0.28
Logistic EuroScore II^27^	4.1 (2.2–7.4)	3.4 (2.2–6.3)	0.63
Mehta score, % risk^32^	0.8 (0.4–1.5)	0.4 (0.3–0.7)	**0.01**

*Note*: Categorical variables are presented as number of patients (%). Continuous variables and clinical risk scores are presented as median (25th–75th percentile) since they commonly failed the Shapiro–Wilk normality test. *p*‐values (in bold type bolded if ≤0.05) are the outcomes of χ^2^‐test (categorical variables) or Mann–Whitney *U*‐tests (continuous variables) for differences between patients who did and did not develop AKI. The estimated glomerular filtration rate (eGFR) was calculated from baseline serum creatinine using the Chronic Kidney Disease Epidemiology Collaboration (CKD‐EPI) equation.^52^

Abbreviations: AKI, acute kidney injury; CABG, coronary artery bypass graft; LVEF, left ventricular ejection fraction; NYHA, New York Heart Association Heart Failure Classification^53^; SCR, serum creatinine.

#### 3.1.2 Clinical parameters measured throughout cardiac surgery

**FIGURE 2:**
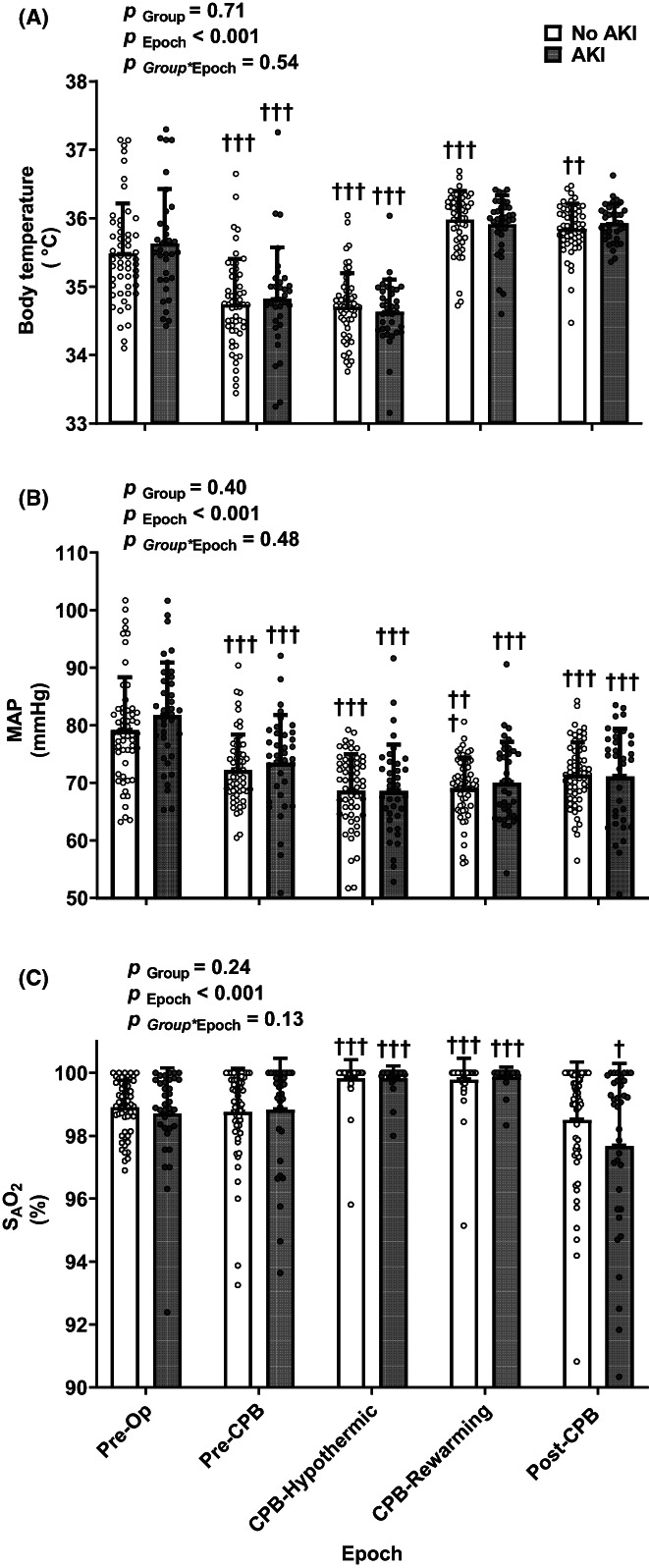
Clinical parameters measured throughout cardiac surgery in the observational study: body temperature, mean arterial pressure (MAP) and saturation of arterial haemoglobin with oxygen (S_A_O_2_). All clinical parameters were averaged across five surgical epochs as indicated on the abscissa. Each symbol represents an individual data point for an individual patient who either did (closed circles) or did not (open circles) develop AKI. Columns and error bars represent mean ± standard deviation (SD) for *n* = 57 (No AKI) and *n* = 36 (AKI) except for body temperature, for which seven patients were excluded from the analyses due to multiple missing values in both groups (total *n* = 86): *n* = 53 (no AKI) and *n* = 33 (AKI). Thus, because conditional mean imputation could not be applied, the entire patient was excluded from the analysis. The surgical procedure was divided into five epochs: Pre‐Op, the preoperative period before the first incision but after induction of anaesthesia; Pre‐CPB, the period of surgery before commencement of cardiopulmonary bypass; CPB‐Hypothermic, hypothermic cardiopulmonary bypass; CPB‐Rewarming, rewarming on cardiopulmonary bypass; and Post‐CPB, the period of surgery after weaning from cardiopulmonary bypass. *p*‐values were derived from a two‐way repeated measures analysis of variance based on the between‐subjects factor group (AKI or no AKI) and the within‐subjects factor time (epoch). *p*‐values for the within‐subjects factor (time) were adjusted using the method of Greenhouse and Geisser.^31^ *p ≤ 0.05, **p ≤ 0.01, ***p ≤ 0.001 for specific comparisons at each epoch between patients who did not develop AKI and patients who developed AKI (Student's unpaired *t*‐test). †*p* ≤ 0.05, ††*p* ≤ 0.01, †††*p* ≤ 0.001 for comparison of each epoch with the first epoch (Pre‐Op) using Student's paired *t*‐test. AKI, acute kidney injury; MAP, mean arterial pressure; S_A_O_2_; oxygen saturation of arterial haemoglobin. Note that ordinate (*y*) axes do not start at zero.

When averaged across all patients with available data, body temperature fell by 0.8 ± 0.1°C from its baseline (preoperative) level during the period before CPB commenced (Figure 2). It remained at a similar level during the period of mildly ‘hypothermic CPB’ but then returned to close to its baseline level during the rewarming period. MAP also fell (by 7.6 ± 1.9 mmHg) during the period before CPB commenced. MAP averaged 68.8 ± 7.0 mmHg during hypothermic CPB and 69.7 ± 6.0 mmHg during rewarming and remained at a similar level after separation from CPB. Arterial blood oxygen saturation (S_A_O_2_) increased slightly during CPB but averaged greater than 97% across the entire surgical procedure. The profile of changes in these variables did not differ significantly between patients who did and did not develop AKI.

#### 3.1.3 Clinical parameters measured during cardiopulmonary bypass

**FIGURE 3:**
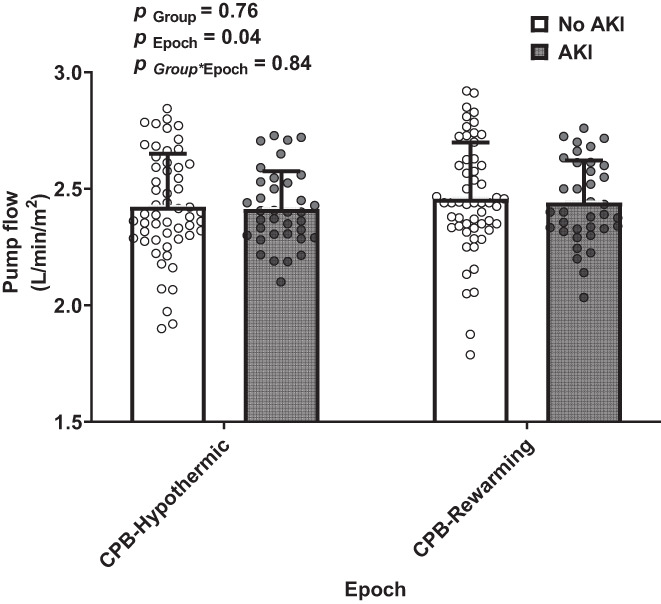
Pump flow during cardiopulmonary bypass in the observational study: pump flow was averaged across the hypothermic and rewarming phases as indicated on the abscissa. Data were missing for two patients ([*n* = 91]: *n* = 55 [no AKI] and *n* = 36 [AKI]). Abbreviations and the format of presentation of the data, including statistical notation, are as for Figure 2. Note that the ordinate (*y*) axis does not start at zero.

When averaged across all patients with available data (*n* = 91), pump flow rose slightly from the hypothermic phase of CPB (2.42 ± 0.20 L/min/m^2^) to the rewarming phase (2.45 ± 0.22 L/min/m^2^). Pump flow averaged across the entire period of CPB was similar in patients who later developed AKI (2.42 ± 0.16 L/min/m^2^) to that in patients who did not develop AKI (2.44 ± 0.22 L/min/m^2^) (Figure 3).

Neither arterial PO_2_ nor blood haemoglobin concentration changed significantly between the hypothermic and rewarming phases of CPB, and both were similar in patients who did and did not develop AKI (Figure S1). However, arterial PCO_2_ increased during rewarming in patients who did not develop AKI (from 38.5 ± 4.8 to 40.5 ± 4.2 mmHg) but did not increase significantly in patients who did develop AKI. Consequently, arterial PCO_2_ was slightly less during rewarming in patients who developed AKI (40.1 ± 2.9 mmHg) than in those who did not develop AKI (41.4 ± 2.9 mmHg).

Systemic DO_2_ was similar in patients who developed AKI compared with those who did not and did not change significantly between the two epochs of CPB (Figure S2). However, with rewarming, systemic VO_2_ increased from 51.1 ± 9.4 mL/min/m^2^ to 60.5 ± 10.5 mL/min/m^2^ when averaged across both groups of patients, mixed venous oxygen saturation fell (from 80.9 ± 3.3% to 77.7 ± 3.4%) and systemic fractional oxygen extraction increased (from 0.19 ± 0.05% to 0.22 ± 0.06%). Nevertheless, these variables remained similar in patients who developed AKI compared with those who did not develop AKI.

#### 3.1.4 Clinical parameters measured before and after cardiopulmonary bypass

At baseline (preoperative), mean pulmonary arterial pressure was greater in patients who later developed AKI (25.4 ± 9.1 mmHg) than those who did not (21.1 ± 6.3 mmHg). Similar differences were seen during the periods before and after CPB (Figure S3). Similarly, central venous pressure was slightly greater during the preoperative period in patients who developed AKI (10.7 ± 3.9 mmHg) than those who did not (8.8 ± 4.5 mmHg). Similar differences were seen during the periods before and after CPB.

## 4 FEASIBILITY CLINICAL TRIAL

### 4.1 Patient characteristics

#### 4.1.1 Recruitment

**FIGURE 4:**
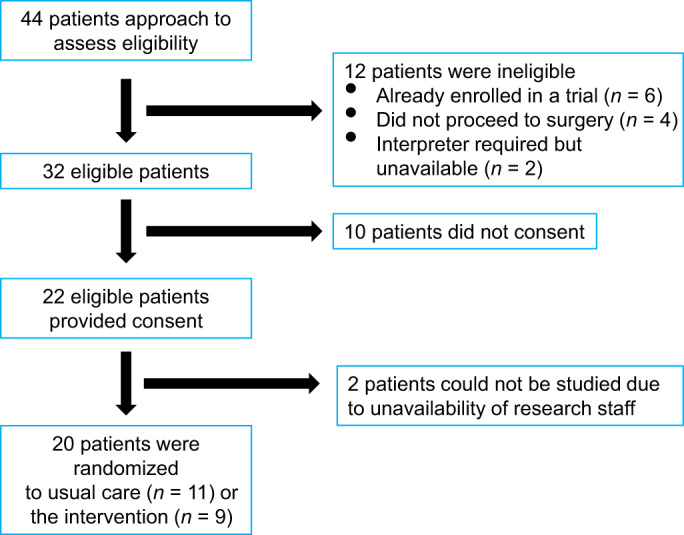
Recruitment of patients: feasibility clinical trial.

Of the 44 patients screened, 12 were ineligible either because they were already enrolled in another trial (*n* = 6) or did not proceed to surgery (*n* = 4) or an interpreter was required to facilitate informed consent and was not available (*n* = 2). Of the 32 eligible patients, 10 did not consent, leaving 22 eligible patients who consented. We could not conduct the trial on two of these patients because of the unavailability of research staff (Figure 4). Preoperative characteristics were similar in the two groups, except that the ejection fraction was greater in the intervention (61.7 ± 6.1%) than in the usual care (51.1 ± 13.7%) group (Table 2). Duration of CPB, but not total aortic cross‐clamp time, was significantly less in the intervention group than in the usual care group.

**TABLE 2:** Preoperative, intraoperative and postoperative characteristics of patients enrolled in the feasibility clinical trial.

Variable	Usual care (*n* = 11)	Intervention (*n* = 9)	*p*
Preoperative characteristics			
Age (years)	68.1 ± 12.4	69.5 ± 9.6	0.79
Male	10 (90.9)	8 (88.9)	0.88
Body mass index, kg/m^2^	28.0 ± 6.3	29.5 ± 4.4	0.55
Body surface area, m^2^	1.94 ± 0.2	2.05 ± 0.19	0.27
CABG	10 (90.9)	6 (66.7)	0.28
Aortic valve replacement	0 (0)	2 (22.2)	0.19
CABG + Valve	1 (9.1)	1 (11.1)	>0.99
Non‐elective	7 (63.6)	3 (33.3)	0.37
Diabetes	3 (27.3)	4 (44.4)	0.64
Myocardial infarction <21 days	7 (63.6)	2 (22.2)	0.09
NYHA I–II	8 (72.7)	8 (88.9)	>0.99
NYHA III–IV	3 (27.3)	1 (11.1)	0.59
Ejection fraction (%)	51.1 ± 13.7	61.7 ± 6.1	**0.05**
Systolic pulmonary arterial pressure (mmHg)	30.8 ± 8.9	28.2 ± 4.2	0.43
Baseline creatinine (μmol/L)	87.0 ± 15.0	81.9 ± 14.2	0.27
EuroScore II (%, median [Q1, Q3])	2.09 (1.14, 7.49)	1.11 (0.87, 1.45)	0.07
Mehta score (%)	0.60 (0.30, 1.65)	0.30 (0.30, 0.90)	0.31
Cleveland score (%)	0.40 (0.40, 0.40)	0.40 (0.40, 0.40)	0.58
Intraoperative characteristics			
Cardiopulmonary bypass time (min)	136 (110, 161)	106 (86, 109)	**0.009**
Cross‐clamp time (min)	127 (68, 130)	75 (64, 97)	0.091
Postoperative characteristics			
Ventilation (hours)	12.6 (6.5, 21.5)	6.5 (5.11, 21.6)	0.43
ICU duration (days)	2.8 (1.08, 3.00)	1.18 (1.00, 2.87)	0.38
Length of stay (days)	8 (7, 12)	7 (7, 11)	0.67
Creatinine change (μmol/L)	20 (10, 28)	33 (5, 36)	0.42
Acute Kidney Injury (KDIGO criteria)	4 (36.4)	5 (55.6)	0.65
90 day survival	11 (100)	9 (100)	>0.99

*Note*: Values are presented as mean ± standard deviation, median (Q1, Q3) or *n* (%). *p*‐values (in bold type bolded if ≤0.05) were derived from an independent two‐sample *t*‐test or Mann–Whitney *U*‐test for continuous variables and from the χ^2^‐test or Fisher's exact test for counts <5 for categorical variables.

Abbreviations: AKI, acute kidney injury; CABG, coronary artery bypass grafting; ICU, intensive care unit; KDIGO, Kidney Disease Improving Global Outcomes; NYHA, New York Heart Association.

#### 4.1.2 Clinical parameters measured during cardiopulmonary bypass

**FIGURE 5:**
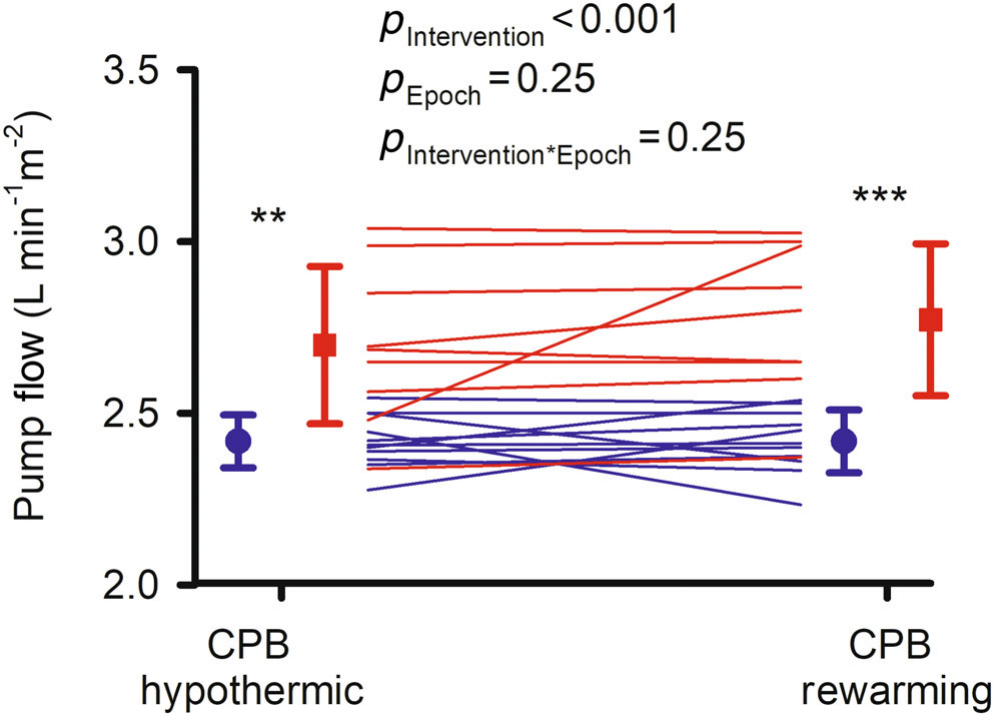
Pump flow during cardiopulmonary bypass in the feasibility clinical trial: lines show data for individual patients in the usual care (blue, *n* = 11) and intervention (red, *n* = 9) groups. Symbols and error bars show mean ± standard deviation. *p‐*values are the outcomes of repeated measures analysis of variance. **p ≤ 0.01, ***p ≤ 0.001 for comparisons between the usual care and intervention groups at each individual epoch (Student's unpaired *t*‐test). Note that the ordinate (*y*) axis does not start at zero.

Pump flow in the usual care group was similar during the hypothermic (2.42 ± 0.08 L min^−1^ m^−2^) and rewarming (2.42 ± 0.08 L min^−1^ m^−2^) phases of CPB (Figure 5). Relative to the usual care group, in the intervention group, pump flow was 12% greater during the hypothermic phase of CPB (2.70 ± 0.23 L min^−1^ m^−2^) and 15% greater during the rewarming phase (2.77 ± 0.22 L min^−1^ m^−2^). The target pump flow of 2.9 L min^−1^ m^−2^ was met in only three of the nine patients in the intervention group. However, pump flow of 2.6 L min^−1^ m^−2^ or greater during hypothermic CPB or rewarming or both was met in eight of the nine patients in the intervention group (89%) but none of the 11 patients in the usual care group. In the case of the one patient in the intervention group in which pump flow did not reach 2.6 L min^−1^ m^−2^, the perfusionist chose not to implement the intervention, stating the opinion that ‘it was too risky and the patient already had suitable perfusion parameters’.

**FIGURE 6:**
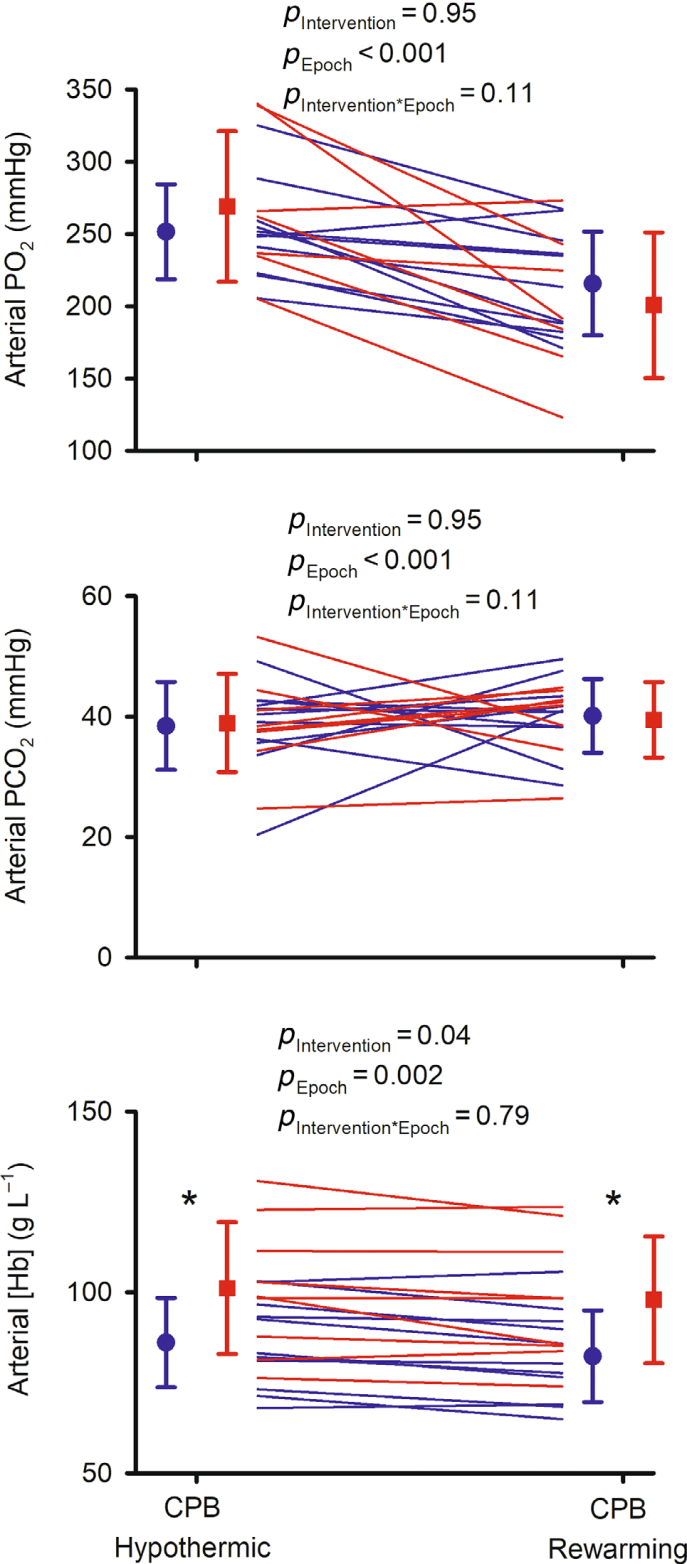
Arterial blood oximetry during cardiopulmonary bypass in the feasibility clinical trial: partial pressure of oxygen in arterial blood (Arterial PO_2_), partial pressure of carbon dioxide in arterial blood (Arterial PCO_2_) and blood haemoglobin concentration (Arterial [Hb]). Lines show data for individual patients in the usual care (blue) and intervention (red) groups. *n* = 11 for the usual care group, with one value imputed for arterial PO_2_. *n* = 9 for the intervention group, except for arterial PO_2_ (*n* = 7), and arterial PCO_2_ (*n* = 8), due to equipment failure. Symbols and error bars show mean ± standard deviation. *p*‐values are the outcomes of repeated measures analysis of variance. *p ≤ 0.05 for comparisons between the usual care and intervention groups at each individual epoch (Student's unpaired *t*‐test). Note that some ordinate (*y*) axes do not start at zero.

During CPB, neither arterial PO_2_ nor arterial PCO_2_ differed significantly between the usual care and intervention groups (Figure 6). However, blood haemoglobin concentration was 18% (hypothermic phase) to 19% (rewarming phase) greater in patients in the intervention group than in the usual care group.

**FIGURE 7:**
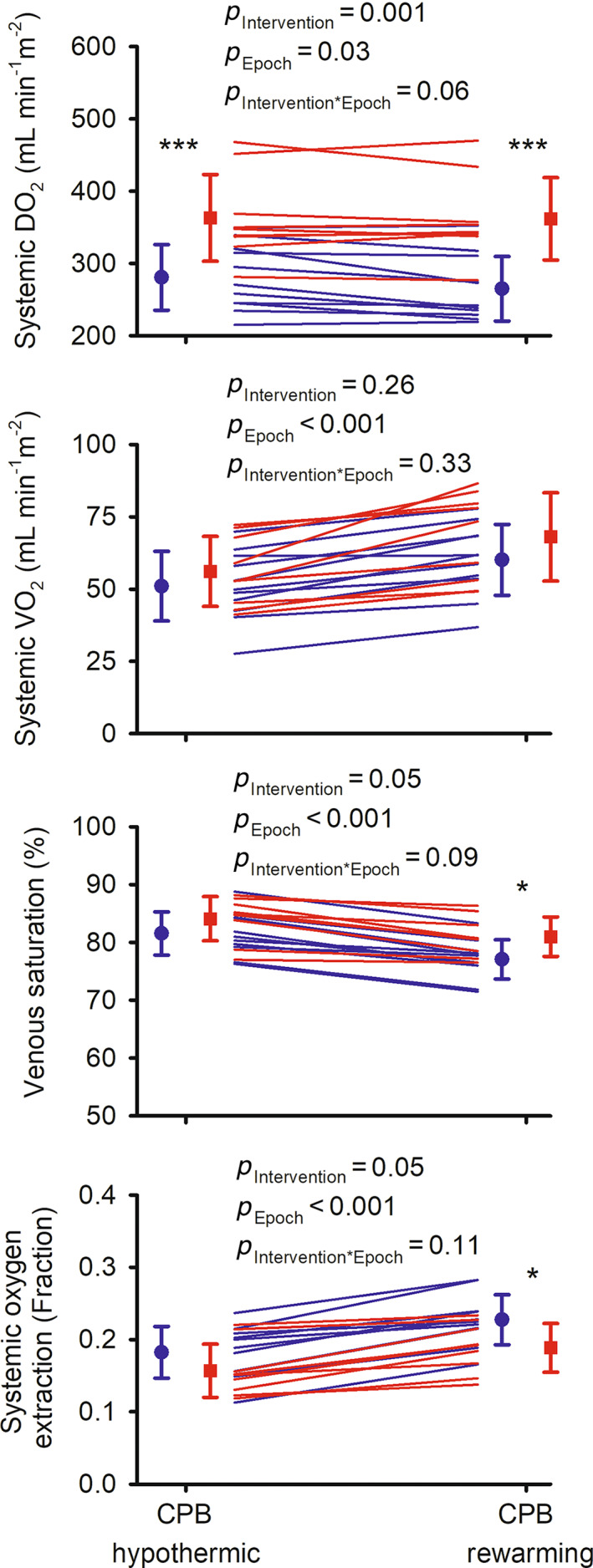
Systemic oxygenation during cardiopulmonary bypass in the feasibility clinical trial: oxygen delivery (DO_2_), oxygen consumption (VO_2_), saturation of venous haemoglobin with oxygen (venous saturation) and fractional extraction of oxygen. Lines show data for individual patients in the usual care (blue) and intervention (red) groups. *n* = 11 for the usual care group and *n* = 9 for the intervention group. Symbols and error bars show mean ± standard deviation. *p*‐values are the outcomes of repeated measures analysis of variance. *p ≤ 0.05, ***p ≤ 0.001 for comparisons between the usual care and intervention groups at each individual epoch (Student's unpaired *t*‐test). Note that some ordinate (*y*) axes do not start at zero.

During CPB, systemic DO_2_ was 29% greater in the intervention group during hypothermic CPB and 37% greater during rewarming on bypass than in the usual care group (Figure 7). In contrast, systemic VO_2_ did not differ significantly between the two groups. Consequently, in the intervention group, fractional oxygen extraction was less (0.19 ± 0.03 for the intervention, 0.23 ± 0.03 for usual care), and mixed venous blood haemoglobin saturation with oxygen greater (81.0 ± 3.4% for the intervention, 77.1 ± 3.4% for usual care), at least during the rewarming phase of CPB.

**FIGURE 8:**
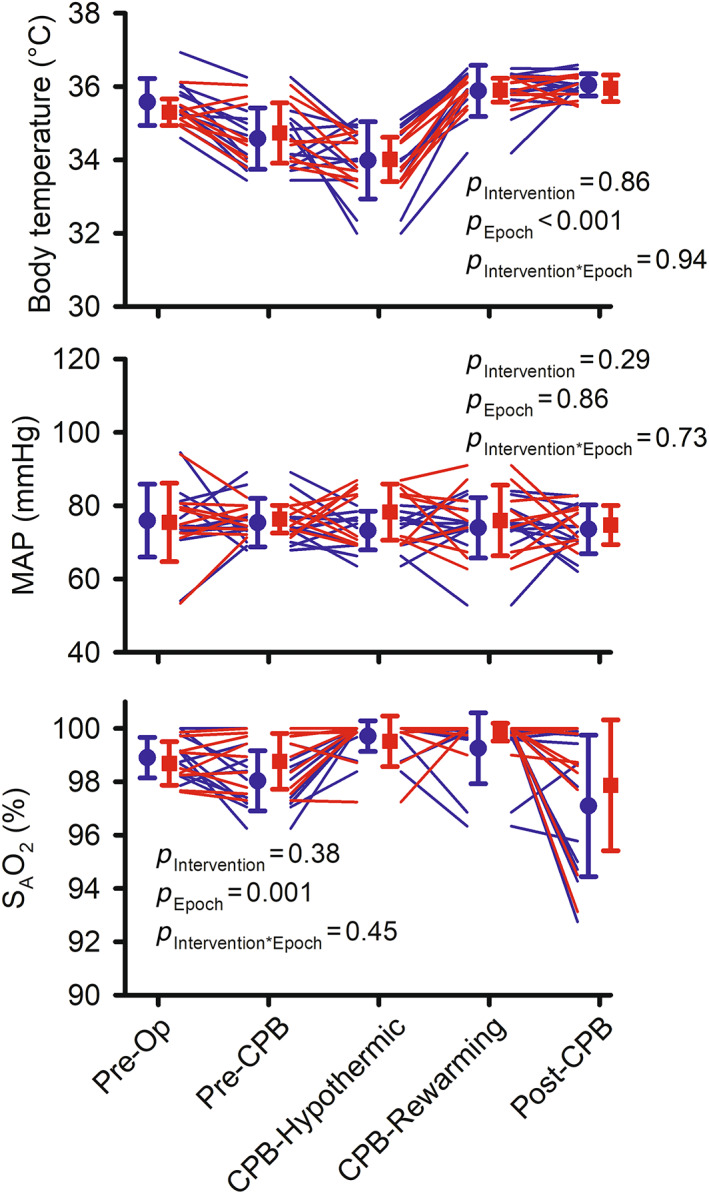
Clinical parameters measured throughout cardiac surgery in the feasibility clinical trial: body temperature, mean arterial pressure (MAP), and saturation of arterial haemoglobin with oxygen (S_A_O_2_). Lines show data for individual patients in the usual care (blue) and intervention (red) groups. *n* = 11 for the usual care group and *n* = 9 for the intervention group. For body temperature, two values were imputed for the usual care group, and one value was imputed for the intervention group. Symbols and error bars show mean ± standard deviation. *p*‐values are the outcomes of repeated measures analysis of variance. Note that ordinate (*y*) axes do not start at zero.

Across all five epochs of surgery, body temperature, MAP and the saturation of arterial haemoglobin with oxygen did not differ significantly between the intervention and usual care groups (Figure 8). However, the target of MAP ≥80 mmHg was achieved, during hypothermic CPB or rewarming or both, in six of the nine patients in the intervention group (66.6%) but only three of the 11 patients in the usual care group (27%).

The combined outcome, of MAP ≥80 mmHg and pump flow of ≥2.9 L min^−1^ m^−2^, during the hypothermic or rewarming phase, was met in two of the nine patients in the intervention group but none of the patients in the usual‐care group.

During the periods before and after CPB, mean pulmonary artery pressure, central venous pressure and end‐tidal CO_2_ did not differ significantly between the intervention and usual care groups (Figure S4).

#### 4.1.3 Blinding

The lead surgeon correctly identified the allocation of the patient to a specific treatment group 73% of the time (11 of 15 responses, *p* = 0.07). Both the lead anaesthetist and lead nurse identified the correct allocation 67% of the time (eight of 12 responses in both cases, *p* = 0.25).

#### 4.1.4 Patient outcomes

Duration of ventilation, ICU stay, hospital stay, postoperative change in serum creatinine concentration, the incidence of AKI and 30 or 90‐day survival did not differ significantly between the two groups of patients (Table 2).

## 5 DISCUSSION

Our current observational findings indicate that hemodynamic management of patients undergoing on‐pump cardiac surgery at our centre, particularly during CPB itself, is relatively homogeneous. Consequently, we were unable to detect systematic differences in pump flow or arterial pressure between those patients who developed CSA‐AKI and those who did not. Thus, at least at the scale of our single‐center study, it is probably not feasible to use observational data to provide a preliminary assessment of whether the use of greater pump flow and/or arterial pressure during CPB can ameliorate the risk of CSA‐AKI. This observation reinforces the notion that randomized clinical trials are required to answer this question. The findings from our feasibility clinical trial indicate that (i) it is feasible to randomize patients to increased pump flow and arterial pressure, (ii) these changes to perfusion conditions are relatively well tolerated by cardiac surgeons, and (iii) albeit based on a very small sample size, the intervention appears to be safe for patients. We also found that it is probably not possible to completely blind members of the surgical team to treatment allocation in such a trial. However, they appear to have a level of uncertainty regarding treatment allocation, which would act to mitigate bias associated with their perceived knowledge of the intervention.

Previous observational studies have provided evidence that low arterial pressure^36^ or pump flow/systemic DO_2_ ^36^, ^37^, ^38^, ^39^ during CPB is associated with a greater risk of CSA‐AKI, although others have failed to detect such associations.^40^, ^41^, ^42^ Notably, all of these previous studies, including those that failed to detect associations of AKI with intraoperative pressure, flow or systemic DO_2_, had larger sample sizes (*n* = 143–920) than the current study. Thus, it remains feasible that a properly designed and powered observational study could provide insight into the impact of pump flow and arterial pressure on the risk of AKI. Regardless, in these previous reports, little attention was paid to the potential for ‘homogeneity of treatment’ (i.e., when standard protocols are followed so that there is little if any between‐patient variation in hemodynamic management during CPB) to limit the effectiveness of the observational approach in this field of research. Furthermore, until recently, these issues have been little investigated in randomized clinical trials.

The Goal Directed Perfusion Trial (GIFT) demonstrated a benefit from the avoidance of systemic DO_2_ <280 mL/min/m^2^.^43^ The incidence of Stage 1 AKI in the intervention arm (11.5%) was close to half that in the usual care arm (22.4%). However, the intervention in the GIFT trial was complex. If systemic oxygen delivery fell below the threshold of 280 mL/min/m^2^, pump flow was increased, but ‘in the event of low hematocrit values and an inability to maintain DO_2_ above the threshold by increasing pump flow, 1 unit of red blood cells was transfused if the venous oxygen saturation was <68% and/or the oxygen extraction rate was >40%’. Thus, the intervention included variable levels of increased pump flow and blood transfusion across the cohort of patients in the intervention arm. There were also no data regarding arterial pressure in their report.^43^ It is also noteworthy that the Transfusion Requirements in Cardiac Surgery‐III (TRICS‐III) trial showed no benefit of a liberal approach to intraoperative blood transfusion with respect to the risk of postoperative AKI.^44^ Thus, there remains an important knowledge gap regarding the impact of pump flow and arterial pressure during CPB on the risk of AKI.

The relative effects of changes in pump flow,^8^, ^19^ arterial pressure,^8^, ^19^ pulsatility of flow and pressure,^19^ blood haemoglobin concentration^45^ and body temperature^46^ on renal oxygenation have recently been assessed in an ovine model of experimental CPB. The overall conclusion from this series of studies was that renal oxygenation, and in particular renal medullary oxygenation, can be improved on bypass by increasing pump flow, arterial pressure or both variables simultaneously but not by increasing blood haemoglobin concentration within a clinically achievable range, or by moderate hypothermia or by partially pulsatile flow. The potential for increased pump flow and MAP to improve renal medullary oxygenation is also supported by recent clinical observations, showing increased urinary PO_2_ at higher target pump flow (3.0 L min^−1^ m^−2^) and MAP (80 mmHg) than at standard targets (2.4 L min^−1^ m^−2^ and 65 mmHg).^20^ These findings provided the impetus for developing a clinical trial of simultaneously increased pump flow and MAP. Our current findings indicate that it is feasible to conduct such a trial.

Approximately one‐third of eligible patients (10 of 32) did not consent to participation in the trial. While this level of recruitment might be viewed as suboptimal, given the difficulties the COVID‐19 pandemic created for the function of elective cardiac surgery in our centre in Melbourne (Australia), we see this level of recruitment as satisfactory. Nevertheless, using other strategies to enhance the consent process may improve recruitment. Alternatively, a cluster randomization approach could be deployed in a multicentre trial. However, this would likely require an increased sample size because of between‐centre differences in patient management.

Fidelity of the intervention, with respect to pump flow, was demonstrated by the fact that pump flow of 2.6 L min^−1^ m^−2^ or greater during hypothermic CPB or rewarming or both was met in eight of the nine patients in the intervention group (89%) but none of the 11 patients in the usual care group. Furthermore, the mean differences in pump flow between the intervention and usual care group, of 0.28 L min^−1^ m^−2^ during the hypothermic phase of CPB and 0.35 L min^−1^ m^−2^ during rewarming on bypass, were highly statistically significant. In the one patient in the intervention group for whom pump flow did not exceed 2.4 min^−1^ m^−2^, the intervention was not instituted due to a deliberate decision by the perfusionist rather than any limit in achievable pump flow (e.g., due to poor venous return). Nevertheless, the actual target of 2.9 L min^−1^ m^−2^ was met in only three of the nine patients in the intervention group. In a recent within‐subject study in which pump flows of 2.4 and 3.0 L min^−1^ m^−2^ were targeted sequentially, the target of 3.0 L min^−1^ m^−2^ was achieved in 17 of the 20 patients.^20^ Thus, it appears to be feasible to achieve higher than standard pump flow in most patients, although achieving a target of 2.9 L min^−1^ m^−2^ may not be feasible in all patients.

One potential concern with an intervention to increase pump flow is the potential need for greater amounts of crystalloid to support the venous return required to achieve greater pump flow, thus leading to haemodilution. We actively discouraged perfusionists from resorting to the addition of excess crystalloid. The success of this measure is indicated by the fact that blood haemoglobin concentration during CPB was, if anything, greater in the intervention group than in the usual care group.

Fidelity of the intervention, with respect to MAP, was demonstrated by the fact that target MAP (≥80 mmHg) was achieved, during hypothermic CPB or rewarming or both, in six of the nine patients in the intervention group (66.6%) but only three of the 11 patients in the usual care group (27%). However, we were unable to detect a significant difference in MAP during CPB between the intervention and usual care groups. In part, this may reflect the small sample size in this feasibility trial. It is also possible that it reflects a change in practice at our centre over the course of our studies, with MAP during the hypothermic phase of CPB averaging 68.8 ± 7.0 mmHg in the observational study conducted from 2015 to 2018 and 73.2 ± 5.3 mmHg in the usual care group for the feasibility clinical trial conducted in 2019 and 2020. It may also reflect some reluctance on the part of the surgical team to expose patients to perceived dangers thought to be associated with elevated MAP during CPB. In a clinical trial of 197 patients, targeting a higher (70–80 mmHg) rather than lower (40–50 mmHg) MAP, albeit with a set pump flow of 2.4 L min^−1^ m^−2^, was found to not be associated with a differing incidence of neurological complications.^47^ However, the higher MAP was found to be associated with lesser regional cerebral oxygenation and more frequent cerebral desaturation as assessed by near‐infrared spectroscopy.^48^ Thus, the optimal level of MAP during CPB remains controversial. Notably, this issue has been dominated by consideration of the cerebral circulation, with little regard for the fact that the lower limit of autoregulation of blood flow is higher for the renal circulation than for the cerebral circulation.^7^, ^49^ Thus, it may be that levels of MAP that provide suitable cerebral perfusion during CPB render the kidney susceptible to ischemia and hypoxia.

The small sample size of our feasibility trial precludes firm conclusions regarding the safety of the intervention of greater than usual pump flow and arterial pressure. Nevertheless, the absence of significant differences in durations of ventilation, ICU stay and hospital stay support its safety. Moreover, all 20 patients in the trial survived for at least 90 days after surgery. We also note that there was no evidence of lesser incidence of AKI or lesser postoperative increase in serum creatinine in the intervention group.

It was not possible to blind perfusionists to treatment allocation. None of the other members of the surgical team was informed of the patient's treatment allocation. However, given their need to monitor the patient's hemodynamic status during the surgical procedure, their blinding could not be assured. Indeed, although not statistically significant, there were trends for the lead surgeon (73%), lead anaesthetist (67%) and lead nurse (67%) to correctly identify the patient's treatment allocation. Thus, it is probably not feasible to effectively blind members of the surgical team from treatment allocation. However, the presence of at least some uncertainty regarding treatment allocation may mitigate the potential for bias associated with presumed knowledge of the intervention.

Both components of our current investigations had limitations. Our observational study had a relatively small sample size (93 patients) and was limited to a single site. Thus, our findings may not be generalizable to other centres. Our feasibility trial had only a small sample size. There was also some heterogeneity with regard to preoperative (ejection fraction) and intraoperative (duration of CPB) characteristics between the two groups. Nevertheless, we were able to demonstrate clear separation in pump flow (but not MAP) between the intervention and usual care groups. We also did not have reliable measurements of preoperative arterial pressure in all patients, so we could not analyse the two datasets with regard to the differences between preoperative and intraoperative arterial pressure, which may be an important determinant of CSA‐AKI.^36^ An additional limitation arises from the fact that we relied on the manual recording of hemodynamic variables, at 5‐min intervals, by a researcher in the operating theatre. Consequently, we likely missed transient changes in these variables that can occur, particularly at the commencement of CPB, at administration of cardioplegia and after removal of the cross‐clamp. However, it is notable that, in the observational study, we identified significant differences in both central venous pressure and mean pulmonary artery pressure between patients who developed AKI and those who did not. Both pulmonary hypertension^50^ and elevated central venous pressure^51^ have been identified previously as risk factors for kidney disease and injury. Thus, our observations regarding these variables provide some confidence in our ability to detect between‐group differences in pump flow and arterial pressure if these were present.

The current feasibility trial provided insights into how the feasibility of a larger trial could be enhanced. We chose to measure urinary oxygen tension in all the patients in the feasibility trial, although we do not report these data herein because the sample size we achieved was too small for meaningful analysis. Nevertheless, this aspect of the protocol restricted our ability to study all eligible patients who consented. Thus, the feasibility of a fully powered trial would be enhanced by lifting this restriction by either not measuring urinary oxygen tension or doing so only in a subset of patients. We also believe recruitment could be enhanced by ensuring that patient consent is sought by a senior medical practitioner or nurse not involved in the patient's care rather than someone introducing themselves as a researcher (as was the case in most instances). There were also several competing trials at our centre at the time of our feasibility trial, which limited recruitment. Taking a multicentre approach could potentially reduce the impact of such competition for patients.

In conclusion, our current findings indicate that the homogeneity of hemodynamic management of adult patients during on‐pump cardiac surgery, at least at our centre, precludes investigation of the associations of postoperative CSA‐AKI with pump flow and MAP during CPB. However, our feasibility clinical trial indicates that it is feasible to undertake a larger‐scale clinical trial to investigate the impact of targeting higher pump flow and MAP on the risk of CSA‐AKI.

## AUTHOR CONTRIBUTIONS

Study concept and design: Roger G. Evans, Andrew D. Cochrane, Julian A. Smith, Amanda G. Thrift, Rinaldo Bellomo and James McMillan. Acquisition, analysis and interpretation of data: Khin M. Noe, Jennifer P. Ngo, Andrew Martin, Michael Z. L. Zhu, Andrew D. Cochrane, Julian A. Smith, Amanda G. Thrift, James McMillan and Roger G. Evans. Drafting of the manuscript: Khin M. Noe, Andrea Don, Michael Z. L. Zhu and Roger G. Evans. Critical revision of the manuscript for intellectual content: Jennifer P. Ngo, Andrew D. Cochrane, Julian A. Smith, Amanda G. Thrift, Rinaldo Bellomo, Andrew Martin and James McMillan. Obtaining funding: Roger G. Evans, Andrew D. Cochrane, Julian A. Smith and Amanda G. Thrift. Statistical analysis: Khin M. Noe and Andrea Don.

## FUNDING INFORMATION

This work was supported by the National Health and Medical Research Council of Australia (GNT1122455) and the National Heart Foundation of Australia (101853 and 102282).

## Supporting information


**Data S1.** supporting Information.

## Data Availability

The data underlying this article will be shared on reasonable request to the corresponding author.
