# Supplementary material for: Intraoperative hemodynamics and risk of cardiac surgery‐associated acute kidney injury: An observation study and a feasibility clinical trial
Source: Clin Exp Pharmacol Physiol. 2023 Aug 7;50(11):878–92. doi: 10.1111/1440-1681.13812 (PMC10947000; doi:10.1111/1440-1681.13812)
Supplement: Supplementary file 1 — Data S1. supporting Information. [file CEP-50-878-s001.docx]

**Online Supplement**

**Intra-operative hemodynamics and risk of cardiac surgery associated acute kidney injury: An observation study and a feasibility clinical trial**

Khin M. Noe^1,2^, Andrea Don^1^, Andrew D. Cochrane^2,3^, Michael Z.L. Zhu^1,2,3^,

Jennifer P. Ngo^1,4*^, Julian A. Smith^2,3^, Amanda G. Thrift^5^, Johnny Vogiatjis^1^,

Andrew Martin^1,2,3^, Rinaldo Bellomo^6,7,8,9^, James McMillan^10^ and Roger G. Evans^1,2,8^

^1^Cardiovascular Disease Program, Biomedicine Discovery Institute and Department of Physiology, Monash University, Melbourne, Australia

^2^Department of Surgery, School of Clinical Sciences at Monash Health, Monash University, Melbourne, Australia.

^3^Department of Cardiothoracic Surgery, Monash Health, Monash University, Melbourne, Australia

^4^Department of Cardiac Physiology, National Cerebral and Cardiovascular Center Research Institute, Osaka, Japan

^5^Department of Medicine, School of Clinical Sciences at Monash Health, Monash University, Melbourne, Australia.

^6^Department of Critical Care, University of Melbourne, Melbourne, Australia

^7^Department of Intensive Care, Austin Health, Heidelberg, Victoria, Australia.

^8^Pre-clinical Critical Care Unit, Florey Institute of Neuroscience and Mental Health, University of Melbourne, Melbourne, Victoria, Australia.

^9^Australian and New Zealand Intensive Care research Centre, Monash University, Melbourne, Australia

^10^Perfusion Services Pty Ltd, Melbourne, Australia

*Deceased

**Author for correspondence:**

Roger Evans, PhD

Department of Physiology

26 Innovation Walk, Monash University, Victoria 3800, Australia

Tel: 61 3 9905 1466; Email: [Roger.Evans@monash.edu](mailto:Roger.Evans@monash.eduroger)


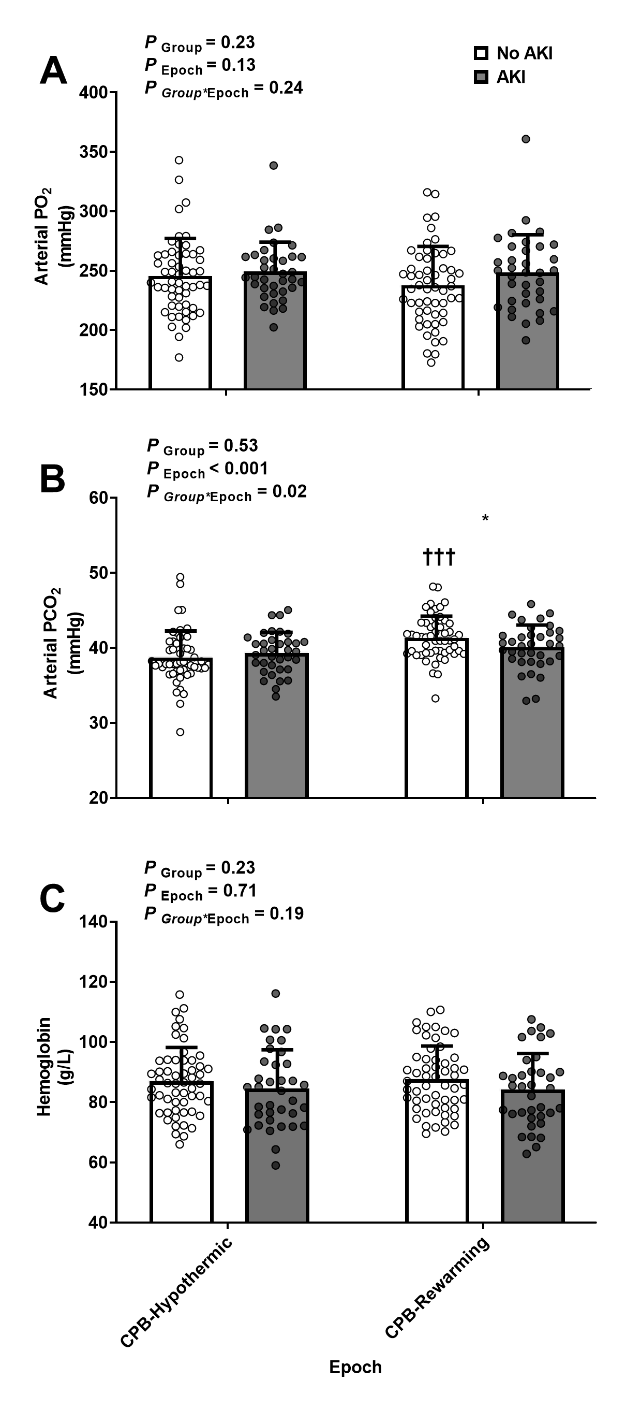


**Figure S1: Arterial blood oximetry during cardiopulmonary bypass in the observational study:** Partial pressure of oxygen in arterial blood (Arterial PO_2_), partial pressure of carbon dioxide in arterial blood (Arterial PCO_2_), and blood hemoglobin concentration (Hemoglobin). All parameters were averaged across two epochs as indicated on the abscissa. Data were missing for arterial PO_2_ for two patients ((n=91): n=55 (No AKI) and n=36 (AKI)). Data were missing for arterial PCO_2_ for three patients and one missing value was imputed ((n=90): n=54 (no AKI) and n= 36 (AKI)). Data were missing for hemoglobin for one patient ((n=92): n=56 (no AKI) and n= 36 (AKI)). Abbreviations and the format of presentation of the data, including statistical notation, is as for Figure 2. Note that ordinate (y) axes do not start at zero.


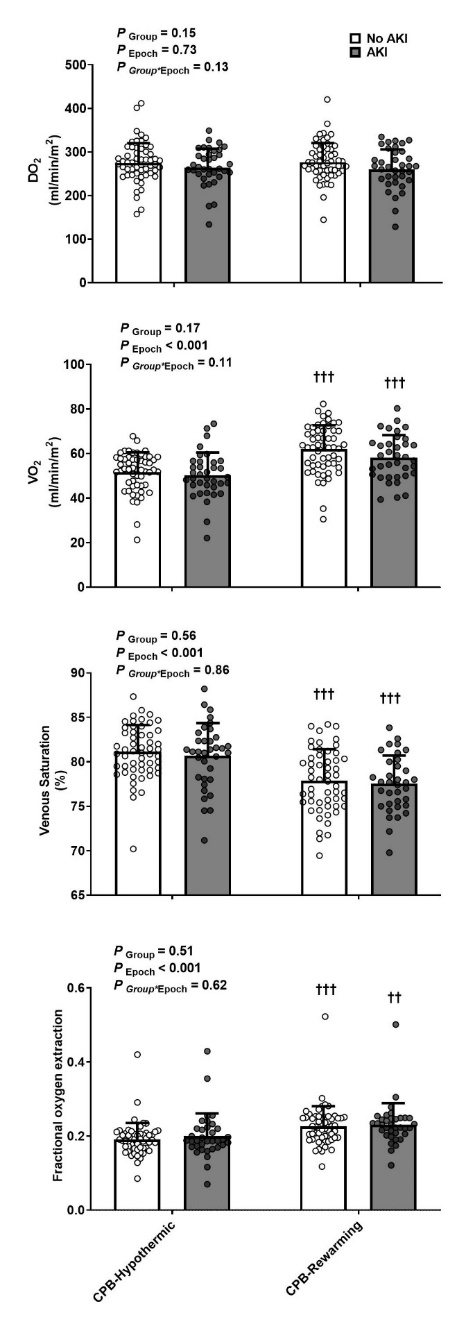


**Figure S2: Systemic oxygenation during cardiopulmonary bypass in the observational study:** Oxygen delivery (DO_2_), oxygen consumption (VO_2_), Saturation of venous hemoglobin with oxygen (venous saturation), and fractional extraction of oxygen. All parameters were averaged across two periods as indicated on the abscissa. Data were missing for DO_2_ and VO_2_ for one patient (n=92) and one value was imputed: (n=56 (no AKI) and n=36 (AKI)). Data were missing for venous saturation for four patients ((n=89): n=54 (no AKI) and n= 35 (AKI)). Data were missing for fractional oxygen extraction for two patients (n=91) and one value was imputed: (n=56 (no AKI) and n= 36 (AKI)). Abbreviations and the format of presentation of the data, including statistical notation, is as for Figure 2. Note that some ordinate (y) axes do not start at zero.


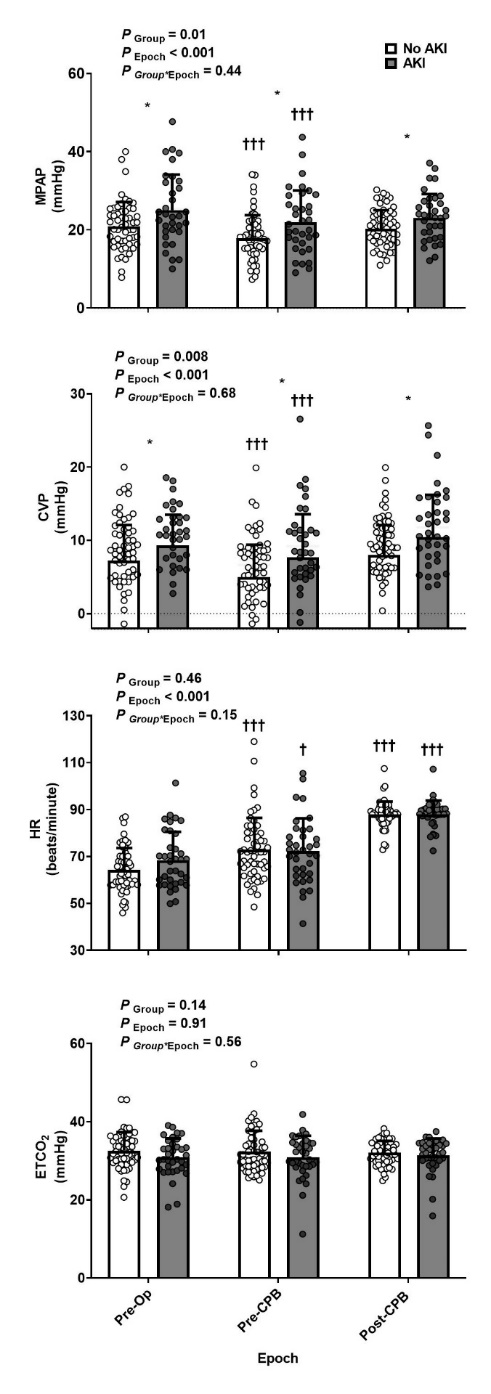


**Figure S3 Clinical parameters measured before and after cardiopulmonary bypass in the observational study:** Mean pulmonary arterial pressure (MPAP), central venous pressure (CVP), heart rate (HR), and end-tidal carbon dioxide partial pressure (ETCO_2_). All parameters were averaged across three periods as indicated on the abscissa. Data were missing for MPAP for seven patients ((n=87): n= 33 (AKI) and n=54 (No AKI)). Data were missing for CVP for four patients ((n=89): n= 34 (AKI) and n= 55 (No AKI)). All 93 patients were included in analyses of HR and ETCO_2_ (n= 36 (AKI) and n= 57 (No AKI)). Abbreviations and the format of presentation of the data, including statistical notation, is as for Figure 2. Note that some ordinate (y) axes do not start at zero.


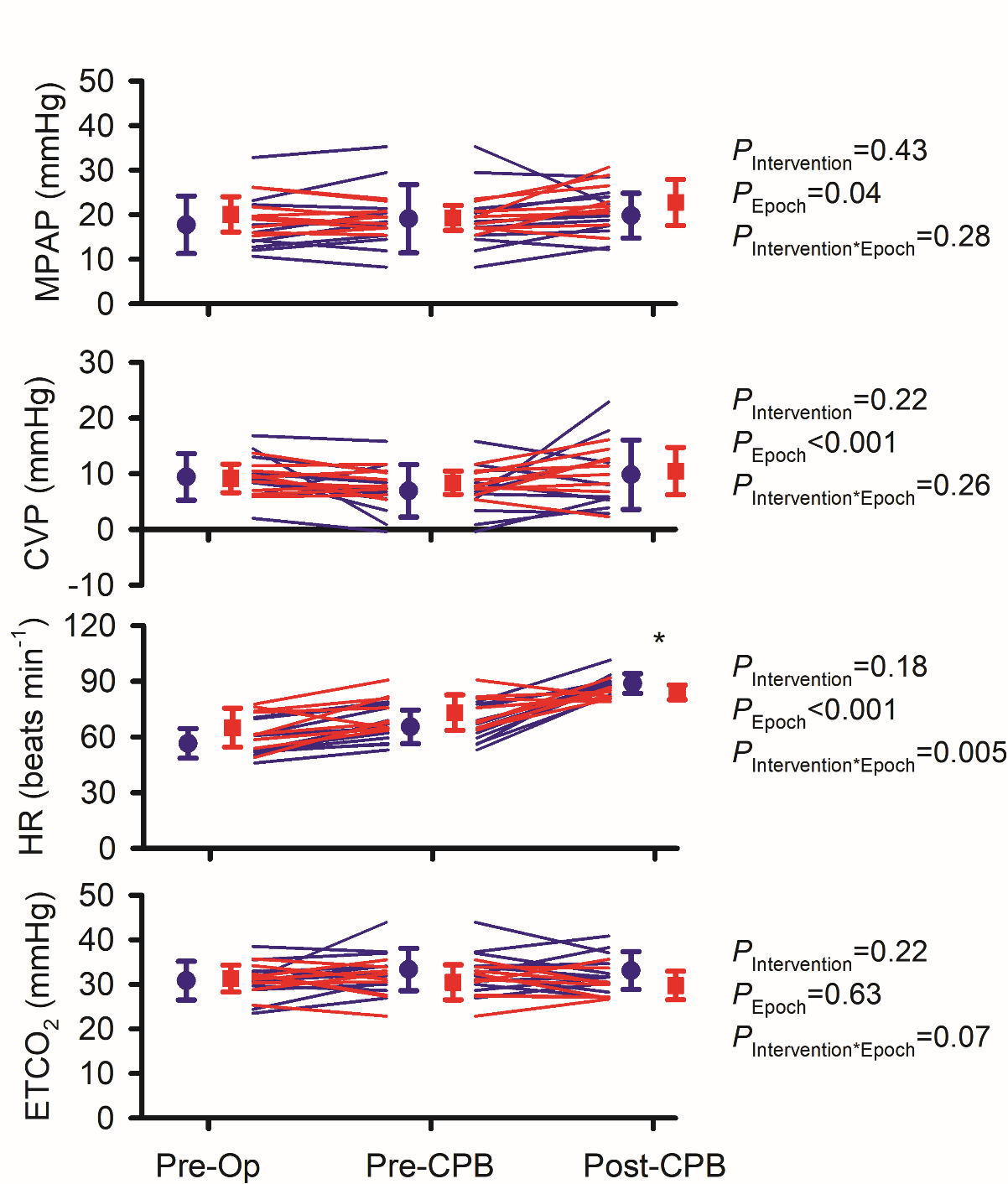


**Figure S4: Clinical parameters measured before and after cardiopulmonary bypass in the feasibility clinical trial:** Mean pulmonary arterial pressure (MPAP), central venous pressure (CVP), heart rate (HR), and end-tidal carbon dioxide partial pressure (ETCO_2_). Lines show data for individual patients in the usual care (blue) and intervention (red) groups. n = 11 for the usual care group, and n = 9 for the intervention group. For CVP, one value was imputed for both the usual care and intervention groups. For ETCO_2_, two values were imputed for the usual care group. Symbols and error bars show mean ± standard deviation. *P* values are the outcomes of repeated measures analysis of variance. **P* ≤ 0.05 for comparisons between the usual care and intervention groups at each individual epoch (Student’s unpaired t-test).
